# High risk genotype distribution of human papillomavirus (HPV) according to age groups in Iranian asymptomatic men

**DOI:** 10.1186/s13027-020-00296-6

**Published:** 2020-05-06

**Authors:** Mehrdad Davarmanesh, Seyed Mohammad Jazayeri, Mehrouz Dezfulian, Mohammad Javad Gharavi

**Affiliations:** 1 Department of Microbiology, Karaj Branch, Islamic Azad University, Karaj, Iran; 2 Research Center for Clinical Virology, Tehran University of Medical Sciences, Tehran, Iran; 3 Medical Genetic Laboratory, Laleh Hospital, Tehran, Iran; 4 Faculty of Paramedicine, Department of Laboratory Sciences, Iran University of Medical Sciences, Tehran, Iran

**Keywords:** Human papilloma virus (HPV) genotypes, HPV in men, Anogenital cancers

## Abstract

**Background:**

The Human Papillomavirus (HPV) is one of the most common sexually transmitted viruses worldwide. HPV infection in men is a serious clinical issue as they could be considered as a reservoir for inadvertently transmitting infection to women. Moreover, genital HPV infection could be a source for anogenital cancers in men.

**Methods:**

This cross sectional study was conducted from January 2017 to December 2018. Four hundred fifteen asymptomatic men who were visited by specialists, referred to Nilou laboratory in terms of high risk (HR) HPV test testing. HR-HPV genotypes were detected using an approved assay which could discover HPV 16, HPV 18 and a pool of other high risk HPV genotypes as well as 16+ other HR and 18 + other HR (as multiple genotypes). SPSS software was used for statistical analysis.

**Results:**

The mean age was 33 ± 8.14 years. Specimens were referred to the laboratory by urologists, (*n* = 132, 32%, 95%CI: 25.0–39.4), dermatologists, (*n* = 104, 25, 95% CI: 19.1–30.9), gynecologists, (*n* = 75, 18, 95%CI: 13.3–29.3) and other specialists (*n* = 104, 25, 95% CI:19.1–30.9). The overall prevalence of other HR HPV, HPV16, HPV18 and multiple genotypes were 54.2% (45/83), 25.3% (21/83), 3.6% (3/83) and 16.8% (14/83), respectively. The frequency of HR-HPV, HPV16 and HPV18 genotypes was the highest among 30–40 years old.

**Conclusion:**

The prevalence of HR-HPV infection among Iranian asymptomatic males was relatively high. Investigation on HPV infection in men as reservoir and transmission vehicle of HPV in addition to screening in women will improve the national public health provisions and will contribute to the application of infection control measurements at a national level.

## Background

Human papillomavirus (HPV) is one of the most common sexually transmitted viruses worldwide [1–3]. World Health Organization (WHO) estimates that the global prevalence of HPV infection is between 9 and 13% or about 630 millions. Persistent infection with HPV is a well-established cause of cervical cancer and there have been massive advancements in the characterization of the natural history of cervical HPV infection in females [4–8]. Although HPV infection in men may be associated with low mortality and morbidity, investigation remains crucial due to its association with genital warts, penile, anorectal, and oropharyngeal cancers as well as to the role of men in HPV transmission to their female sexual partners [9–11]. It is estimated that the prevalence of HPV-infection is 20% of all men, reaching 70% in some age groups, especially among individuals between 15 and 24 years of age [9]. However, compared with cervical HPV infection, relatively little is known about the epidemiology of HPV infection in men [12]. In fact, it has been suggested that men may constitute a reservoir for inadvertently transmitting infection to women and because of its asymptomatic nature, therefore, contributing to the persistence of infection and cancer [13]. Conversely, unlike women, there are no guidelines for screening of males and no any other regular HPV testing does exist. Information about HPV transmission probabilities in men is of paramount importance to evaluate the impact of prophylactic vaccines against HPV and to monitor the distribution of specific types before and after the introduction of HPV vaccine [14]. Global data on age-specific prevalence of human papillomavirus (HPV) infection in males, especially for oncogenic HPV types 16 and 18, are essential for future efforts to prevent HPV-related diseases, including expanded access to HPV prophylactic vaccines for boys and young men [15, 16]. Since HPV types 16 and 18 have been designated as conferring the greatest risk for cervical disease, their detection may prove useful in guiding patient management [17]. In this regard, the difference in age patterning of different HPV-related diseases suggests that genital HPV natural history in men varies by age and HPV type. The objective of the current survey was to characterize the type-specific HPV genital infection positivity distribution in men according to age in order to identify age ranges and genotypes that can directly endanger asymptomatic men who referred to Nilou laboratory and consequently, their partners.

## Methods

### Study population

This cross sectional study was conducted from January 2017 to December 2018. Four hundred and fifteen asymptomatic heterosexual males who were mostly visited by dermatologists, gynecologists and urologists referred to Nilou laboratory in order to test HR-HPV because of importance of detecting infected men (HR-HPV+) as reservoir which can endanger women as their sexual partners. A written informed consent guaranteeing confidentiality was taken from each patient. The study was approved by the National Ethics Committee for Biomedical Research affiliated to The Ministry of Health and Medical Education. Demographic data were recorded by trained laboratory staff.

### Sampling and HPV testing

Urogenital specimens were taken from the urinary meatus, frenulum, glans of penis and scrotum using dry polyester tipped or dry foam swabs. To detect HR-HPV genotypes in patient specimens, Cobas®4800 (Roche Molecular Systems, CA, USA) was applied using oligonucleotide probes labeled with four different fluorescent dyes. The Cobas®4800 HPV test included fully automated sample preparation combined with real-time PCR technology and software that integrated the two modules. One milliliter aliquots of liquid specimen were transferred to 13 mL barcoded tubes provided by the manufacturer. The Cobas®4800 HPV test was performed according to the manufacturer’s protocol. The amplified signals from 12 high-risk HPV types (31, 33, 35, 39, 45, 51, 52, 56, 58, 59, 66 and 68), were detected using the same fluorescent dye, while HPV16, HPV18 and beta-globin signals were each detected with their own appropriate fluorescent dye. COBAS®4800 generates individual qualitative results for: HPV 16, HPV 18 and a pool of other high risk HPV genotypes as well as 16**+** other HR and 18 **+** other HR (as multiple genotypes).

### Statistics

This study shows the distribution of categorical variable as HR-HPV genotype in men of different age groups. The distribution of nominal variable in different groups was tested using Cramer’s V test. Proportions were reported with 95% confidence interval. *P*-value was calculated by using SPSS software version 22 and it was considered significant when < 0.05.

## Results

A total of 415 men referred to Nilou medical laboratory for checking high- risk HPV were included in this survey. The mean age was 33 ± 8.14 years. The number of men that referred to the laboratory in the order of young to older ages were as follows: < 30 years, 120 (28.9, 95% CI: 24.6–33.0, *P* value 0.542); 30–40 years, 214 (51.6, 95% CI: 24.6–33.0, *P* value 0.571); 40–50 years, 61 (14.7, 95% CI: 11.3–18.3, *P* value 0.139) and > 50 years, 20 (4.8, 95% CI: 2.9–6.7, *P* value 0.656) (Table 1). Results from patients file revealed that specimens were referred to the laboratory by urologists, (*n* = 132, 32, 95% CI: 25.0–39.4), dermatologists, (*n* = 104, 25, 95% CI: 19.1–30.9), gynecologists, (*n* = 75, 18, 95% CI: 13.3–29.3) and other specialists, (n = 104, 25, 95% CI: 19.1–30.9) (Table 2). Overall HR- HPV prevalence was 20%. The prevalence of other HR HPV, HPV16, HPV18 and multiple genotypes were 54.2% (45/83), 25.3 (21/83), 3.6% (3/83) and 16.8% (14/83) respectively (Table 1). The HR-HPV distribution according to age group was revealed in the following order: for < 30 years old, of 17 samples, 8 (47%), 5 (29%), 4 (32.5%) with prevalence of 1.9% (95% CI: 0.7–3.6), 1.2% (95% CI: 0.2–2.0) and 0.9% (95% CI: 0.1–1.7) were belonged to other HR, HPV16 and multiple genotypes, respectively (Table 3). For 30–40 years old, out of 51 samples, 28 (55%), 13 (25%), 3 (6%) and 7 (13.7%) with prevalence of 6.7% (95% CI: 4.5–9.1), 3.1% (95% CI: 1.4–5.0), 0.7% (95% CI: 0.1–1.3) and 1.7% (95% CI: 0.4–3.0) were contained other HR, HPV16, HPV18 and multiple genotypes, respectively (Table 3). For 40–50 years old, of 11 samples, 6 (55%), 3 (27%), 2(18.1%) with prevalence of 1.4% (95% CI: 0.4–2.4), 0.7% (95% CI: 0.1–1.3) and 0.5% (95% CI: 0.0–1.0%) were positive for other HR, HPV16 and multiple genotypes, respectively (Table 3). Finally, for > 50 years old, of 4 samples, 3 (75%) and 1 (25%) with prevalence of 0.7% (95% CI 0.1–1.3) and 0.2% (95% CI 0.0–0.6) were belonged to other HR and multiple genotypes, respectively (Table 3). Prevalence of HPV16 according to age was 1.2, 3.1 and 0.7% for < 30, 30–40 and 40–50 years old, respectively (Table 3)*.* Only men in the group of 30–40 years old showed to be positive for HPV18 (Table 3). Prevalence of HR HPV infections varied according to age ranging from a maximum of 6.7% in other HR types at age 30–40 to a minimum of 0.2% in multiple types at age > 50 (Table 3)*.* The HR-HPV infection was most prevalent in the group of age between 30 and 40 years old with a rate of 3.1% for HPV16, 6.7% for the other HR HPV types and 1.7% for multiple HPV infection (Table 3). The calculated *P*-value was 0.68 for correlation intensity between HPV genotypes and age groups.

**Table 1:** Demographic and HPV genotypic characteristics of patients

Characteristic	All sample n (%)	*P* value	CI 95% (for Age and HPV ± genotype)
	415 (100)	α =0.05	Lower - Upper
Age			
< 30	120 (28.9)	0.542	24.6–33.0%
30–40	214 (51.6)	0.571	47.0 –56.6%
40–50	61 (14.7)	0.139	11.3–18.3%
> 50	20 (4.8)	0.656	2.9–6.7%
HPV Genotyping Results			
Negative	332 (80.0)	< 0.005	76.4–83.8%
Positive (Total)	83 (20)	< 0.005	16.4–23.9%
16	21 (25.3)	0.043	16.9–34.9%
18	3 (3.6)	_ a	0.0–8.4%
Other HR	45 (54.2)	0.092	44.6–62.1%
Multiple^b^	14 (16.8)	0.013	9.6–25.3%

^a^Value could not be calculated

^b^16 + Other HR,18+ Other HR,16 + 18

**Table 2:** Distribution of specialists that referred men to Nilou lab (*N* = 415)

Specialist	Frequency	(%)	95% Confidence Interval	
			Lower	Upper
Urologist	132	32	25.0	39.4
Dermatologist	104	25	19.1	30.9
Gynecologist	75	18	13.3	23.9
Other	104	25	19.1	30.9
**Total**	415	100.0	100.0	100.0

**Table 3:** Characteristic of HPV genotypes Frequency according to age groups and Prevalence of HPV infection among men (*N* = 415)

Age	N^**a**^	HPV genotype	HPV + genotype Frequency	HPV + genotype (%)	No.HPV types	Prevalence (95%CI) (***N*** = 415)
**< 30**	**120**	Other HR 16 Multiple^b^	8 5 4	47.0 29.4 32.5	8 5 4	1.9% (0.7–3.6%) 1.2% (0.2–2.0%) 0.9% (0.1–1.7%)
		**Total**	**17**	100	**17**	**4.0 (2.4–6.2%)**
**30–40**	**214**	Other HR 16 18 Multiple	28 13 3 7	54.9 25.4 5.9 13.7	28 13 3 7	6.7% (4.5–9.1%) 3.1% (1.4–5.0%) 0.7% (0.1–1.3%) 1.7% (0.4–3.0%)
		Total	**51**	100	**51**	**12.2 (9.1–15.1%)**
**40–50**	**61**	Other HR 16 Multiple	6 3 2	54.5 27.2 18.1	6 3 2	1.4% (0.4–2.4%) 0.7 (0.1–1.3%) 0.5% (0.0–1.0%)
		Total	**11**	100	**11**	**2.6%(1.2–4.3%)**
**> 50**	**20**	Other HR Multiple	3 1	75.0 25.0	3 1	0.7% (0.1–1.3%) 0.2% (0.0–0.6%)
		Total	**4**	100	**4**	**0.9% (0.1–1.7%)**
**Total 415**			**Total 83**			

^**a**^ Number of age groups

^b^16 + Other HR,18+ Other HR,16 + 18

## Discussion

Sexual behavior characteristics in both women and men are of paramount impacts regarding HPV infection and transmission. Therefore, sexual behavior of males can influence the risk of HR-HPV infection in their sexual partners. The presence of HPV infection in men is largely unknown. Although men are regarded as a dominant factor in infection transmission to their female sexual partners, they frequently do not develop clinically significant HPV-related lesions and are usually asymptomatic during relatively short lasting infections [18]. An important issue to note is that, the infected males are not aware of the disease due to the absence of symptoms that endanger both themselves and their sex partners leading to the risk for HPV-related diseases including cancers. The prevalence of HR-HPV infection among asymptomatic men in previous studies were included in 39, 14.3, 8.7, 20 and 9.5% in Croatia [19], Poland [18], Mexico [14], Lithuania [20] and Iran [4] respectively (Table 4). The prevalence of genital HPV infections in men varies widely due to differences in the populations studied, sampling sites, HPV DNA detection method used, race, age, sexual orientation and circumcision [21].

**Table 4:** Reported HR HPV distribution among referral asymptomatic men from different surveys

Author/ Year	Country	HR-HPV genotype prevalence			
		Total (%)	16 (%)	18 (%)	Other HR (%)
Jersovein et al. [20]/2019	Lituania	20	15	0	70
Bosnjak Z et al. [19]/2013	Croatia	39	17.8	1.3	28.2
Parada et al. [14] /2010	Mexico	8.7	13.6	15.9	70.4
Dutkiewiczet al [18]./2013	Poland	14.3	14.4	_^a^	_
Salehi-Vaziri et al. [4]/2015	Iran	9.5	23.9	19.5	63
Present Study/2019	Iran	20	25.3	3.6	54.2

^a^ Genotype prevalence could not be written

In our study, HR-HPV was highly prevalent, with 20% of men testing positive for HR-HPV (Table 1). These results were among the high range of HR-HPV prevalence from above-mentioned regions. Moreover the present study showed that of overall HR-HPV prevalence, the frequency of other HR HPV, HPV16 and HPV18 were 54.2, 25.3 and 3.6%, respectively. On the other hand Salehi-Vaziri et al. in 2015 reported a prevalence of HR-HPV in 9.5% of Iranian men referred to the Sexually Transmitted Infections (STI) clinics showing a prevalence of HR HPV, HPV16 and HPV 18 were 63, 23.9 and 19.5% (Table 4).

Therefore, unlike our report, their survey showed that HPV18 was more prevalent among Iranian males. The prevalence of multiple genotypes was also comparable among ours and Salehi-Vaziri studies, 16.8 and 8.7% respectively (results not shown). These differences could be related to the sampling bias referred to STI clinic and the difference between HPV genotype detection methods. The results of both studies showed that the more vulnerable age groups for HR HPV were young males between of 30 and 40 years old. Age group > 50 showed the lowest prevalence of HR-HPV, however, the small sample size in this group avoid us to draw a definite conclusion. Overall correlation intensity between HPV genotypes and age groups was not significant (*P*-value = 0.68).

There are not too many reports in terms of men referral system from specialist doctors to medical laboratories. Our study showed that urologists (32%), dermatologists (25%) and gynecologists (18%) had a higher percentage in referring men to the Nilou laboratory for HPV testing. From public health point of view, this finding can have a significant impact on targeting the most-visiting specialist for getting access to detect infected males across the country.

According to the World Health Organization (WHO) draft published in 2019, the message entitled “global strategy toward the elimination of cervical cancer as public health problem” was considered as a global strategy for comprehensive, population-based approach to encourage all countries for the elimination of cervical cancer. This policy incorporates the vaccination and other public health issues on females. Therefore, recognition of HPV infection in men as a vehicle for HPV transmission in females could impact the implementation of preventive measures for cervical cancer and other HPV-related diseases in both genders. Protective measures like HPV vaccination in men will not only care for them but will also benefit their sexual partners. Therefore, policies to mitigate HPV infections in couples may give rise in public health improvement.

## Conclusion

The results of the current study showed that the prevalence of HR-HPV infection among Iranian asymptomatic males was relatively high. Therefore, a survey of HPV infection in men as reservoir and transmission vehicle of HPV in addition to screening in women will improve the national public health provisions and will contribute to the application of infection control measurements at a national level.

## Data Availability

Authors can confirm that all relevant data are included in the article and materials are available on request from the authors.
